# Visible Light Optical Coherence Tomography (OCT) Quantifies Subcellular Contributions to Outer Retinal Band 4

**DOI:** 10.1167/tvst.10.3.30

**Published:** 2021-03-26

**Authors:** Tingwei Zhang, Aaron M. Kho, Glenn Yiu, Vivek J. Srinivasan

**Affiliations:** 1 Department of Biomedical Engineering, University of California Davis, Davis, California, USA; 2 Department of Ophthalmology and Vision Science, University of California Davis, Davis School of Medicine, Sacramento, California, USA; 3 Department of Ophthalmology, New York University Langone Health, New York, New York, USA; 4 Department of Radiology, New York University Langone Health, New York, New York, USA; 5 Tech4Health Institute, New York University Langone Health, New York, New York, USA

**Keywords:** optical coherence tomography, retinal pigment epithelium, Bruch's membrane, retina

## Abstract

**Purpose:**

To use visible light optical coherence tomography (OCT) to investigate subcellular reflectivity contributions to the outermost (4th) of the retinal hyperreflective bands visualized by current clinical near-infrared (NIR) OCT.

**Methods:**

Visible light OCT, with 1.0 µm axial resolution, was performed in 28 eyes of 19 human subjects (21–57 years old) without history of ocular pathology. Two foveal and three extrafoveal hyperreflective zones were consistently depicted within band 4 in all eyes. The two outermost hyperreflective bands, occasionally visualized by NIR OCT, were presumed to be the retinal pigment epithelium (RPE) and Bruch's membrane (BM). RPE thickness, BM thickness, and RPE interior reflectivity were quantified topographically across the macula.

**Results:**

A method for correcting RPE multiple scattering tails was found to both improve the Gaussian goodness-of-fit for the BM intensity profile and reduce the coefficient of variation of BM thickness in vivo. No major topographical differences in macular BM thickness were noted. RPE thickness decreased with increasing eccentricity. Visible light OCT signal intensity in the RPE was weighted to the apical side and attenuated more across the RPE in the fovea than peripherally.

**Conclusions:**

Morphometry of the presumed RPE and BM bands is consistent with known anatomy. Weighting of RPE reflectivity toward the apical side suggests that melanosomes are the predominant contributors to RPE backscattering and signal attenuation in young eyes.

**Translational Relevance:**

By enabling morphometric analysis of the RPE and BM, visible light OCT deciphers the main reflectivity contributions to outer retinal band 4, commonly visualized by commercial OCT systems.

## Introduction

Optical coherence tomography (OCT) is a powerful cross-sectional imaging modality that has become a standard of clinical ophthalmic care routinely used in diagnosis and management of ocular diseases.^1^ Although OCT provides cross-sectional images that resemble histological sections of the retina in some respects, reflective structures in OCT may or may not coincide with those visualized by histological staining protocols.^2^ Thus the correct assignment of the OCT bands requires a careful consideration of both anatomy and optical physics. Although the inner retinal layers in OCT match reasonably well with histological sections,^3^ interpretation of the outer retinal hyperreflective bands is less straightforward, in part because of the intimate relationship between the retinal pigment epithelium (RPE) and photoreceptors,^4^^,^^5^ the proximity of the RPE to Bruch's membrane (BM),^6^ and the fine layers of stacked photoreceptor^7^ and RPE^8^ organelles. Changes in the axial positioning and number of outer retinal hyperreflective OCT bands^9^^,^^10^ accompany the transition from a dense cone-only foveola to a rod-dominated periphery with sparse cones.^11^^,^^12^ These findings suggest a critical connection between OCT image banding and outer retinal photoreceptor and RPE anatomy. Encapsulating knowledge in 2014, the International Nomenclature for Optical Coherence Tomography (IN•OCT) Panel designated four outer retinal bands.^13^ Vigorous debate^7^^,^^14^^–^^17^ has centered around the origins of bands 2 and 3, designated as the photoreceptor inner segment ellipsoid zone and the interdigitation zone (IZ), respectively, by the IN•OCT Panel. Although somewhat less contentious, numerous questions about the outermost band 4 assigned to the RPE-BM complex, remain unresolved. The IN•OCT Panel concluded that RPE and BM “are often not separable under normal conditions.” Indeed, models of outer retinal reflectivity based on available NIR OCT technologies neither explicitly examine subcellular reflectivity of the RPE nor do they distinguish between the RPE and BM.^18^ BM and the RPE are occasionally separable in research prototype NIR OCT of normal eyes,^9^^,^^19^^,^^20^ and they are reliably separated by commercial instruments in some pathologies.^21^^–^^23^ Yet, to date, the contributions to the reflectivity of this fourth band have not been conclusively resolved according to anatomic space (e.g., interdigitation zone, apical RPE, basal RPE, and Bruch's membrane) or organelle (e.g., nucleus, mitochondria, lipofuscin, melanosome, melanolipofuscin). Although some acknowledge that melanosomes with melanin are major sources of RPE reflectivity,^8^^,^^24^^–^^27^ this viewpoint is not universal.^16^^,^^28^^,^^29^

Complementary approaches have emerged to augment clinical OCT images and aid in their interpretation. For instance, prototype adaptive optics (AO) OCT instruments with higher transverse resolutions depict the en face details of reflective structures within the clinical OCT bands such as the cone outer segment tips,^30^ as well as the rod outer segment tips and the RPE,^31^^,^^32^ which strongly suggest their underlying cellular origins. Recently, a complementary, ex vivo serial block-face scanning electron microscopic approach was proposed to generate an atlas of organelles, representing candidate reflective structures, in the human RPE.^8^^,^^33^ Yet another ex vivo microscopic approach was recently applied to catalogue the axial distribution of autofluorescent RPE granules.^34^ In vivo imaging of the RPE and BM, with a level of axial detail and definition that matches this available anatomical knowledge, could enhance our understanding of the subcellular contributions to reflectivity of band 4.

OCT axial resolution in air is given by $\delta \lambda =0.44\lambda_{0}^{2}/d\lambda$ where λ_0_ is the central wavelength and *d*λ is the full-width-at-half maximum (FWHM) bandwidth. For a fixed *d*λ, finer axial resolutions are enabled by a transition to shorter wavelengths.^35^ Recently, visible light OCT was developed for ultrahigh-resolution retinal imaging.^36^^,^^37^ With the latest technical advances,^38^^–^^40^ visible light OCT image quality now surpasses that of commercial NIR OCT and state-of-the-art NIR OCT^41^ in some respects.^39^^,^^40^ Besides providing finer axial resolution, visible light enhances the contrast of BM relative to the basal RPE,^25^ providing unique advantages for elucidating outer retinal band 4 compared to commercial NIR OCT. In this study, we used a prototype visible light OCT system with 1.4 µm axial resolution in air (1.0 µm axial resolution in tissue), although without lateral cellular resolution,^42^ to delve into the subcellular axial reflectivity within band 4.

## Methods

### Outer Retinal Bands in Visible Light OCT

**Figure 1.**
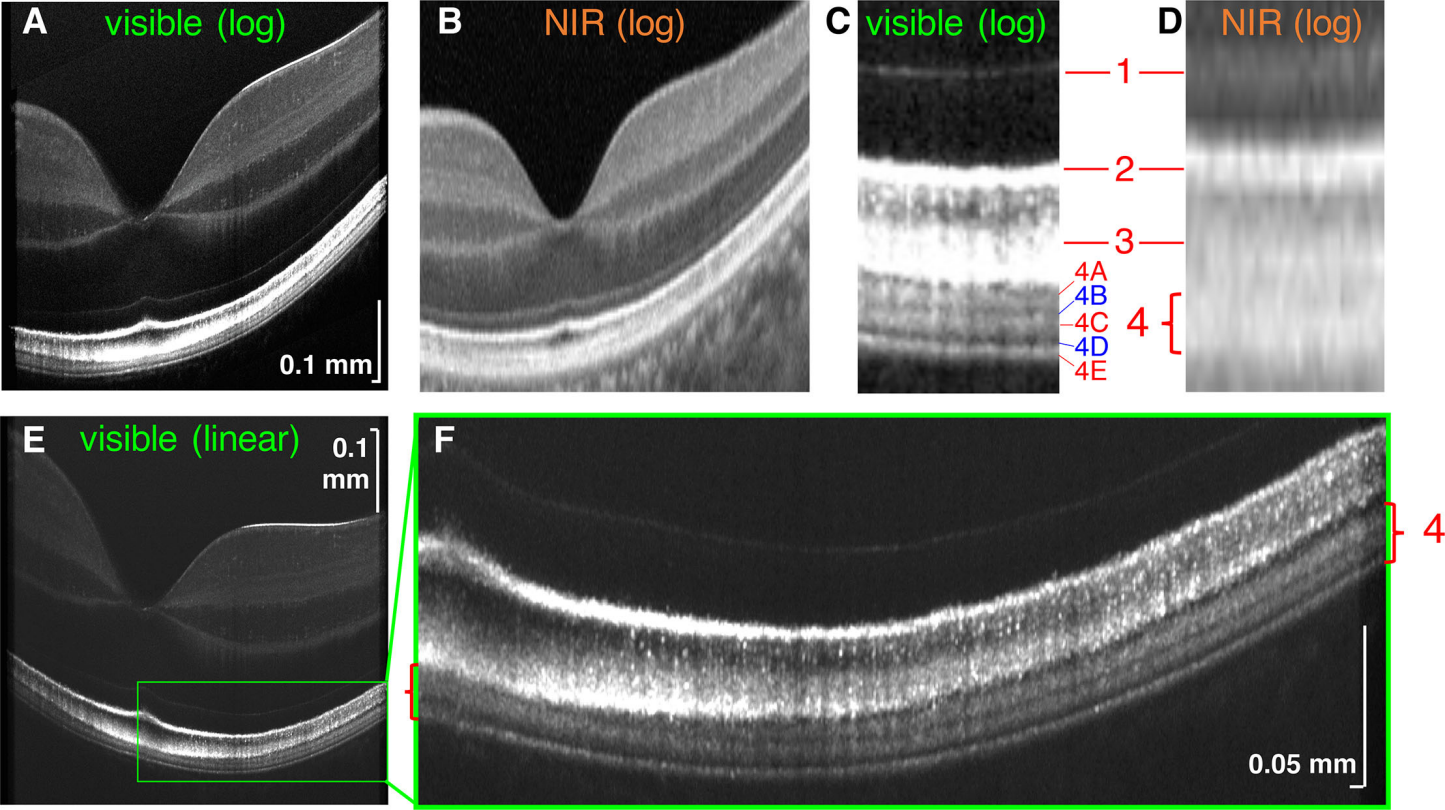
Visible light OCT (**A**) and commercial NIR OCT (cropped enhanced HD line, Optovue) (**B**) of the retina of a 27-year old Asian male (logarithmic scale). Parafoveal visible light OCT magnified view (**C**) shows three hyperreflective bands (4A, 4C and 4E) and two hyporeflective zones (4B and 4D) within the single hyperreflective band 4 observed in commercial NIR OCT (**D**). (**E**, **F**) Linear scale improves the visualization of visible light OCT sublayers within band 4 (*red brackets*) across the macula, showing a clear transition from two hyperreflective bands in the fovea (*left bracket*) to three hyperreflective bands extrafoveally (*right bracket*). NIR, near-infrared; log, logarithmic scale; linear, linear scale.

Figure 1 shows a detailed comparison of visible light OCT (Figs. 1A, 1C) and commercial NIR OCT (Figs. 1B, 1D), with regard to the 4 IN•OCT outer retinal bands.^13^ In the literature, band 1 is universally called the external limiting membrane,^9^^,^^13^^,^^30^^,^^43^ band 2 is alternatively attributed to the photoreceptor inner segment/outer segment junction^9^^,^^30^^,^^43^ or ellipsoid zone,^13^ and band 3 is alternatively attributed to the cone outer segment tips (COST)^9^^,^^19^^,^^43^ or cone IZ.^13^^,^^19^ Because the goal of our study is not to investigate these bands, we avoid taking a direct position on their attribution.

The main topic of the present study, band 4, is typically attributed to the RPE and BM,^13^^,^^19^^,^^43^ with acknowledgement of possible contributions from rod outer segment tips (ROST)^9^^,^^19^^,^^43^ or IZ.^19^ In this work, we adopt the IN•OCT terminology “zone” for OCT features that seem to localize to a particular anatomic region.^13^ Within extrafoveal band 4 of commercial NIR OCT (Figs. 1B, 1D), visible light OCT depicted three hyperreflective zones or bands (denoted as 4A, 4C, and 4E to convey that they are contained within band 4) separated by two hyporeflective zones (likewise denoted as 4B and 4D). In the cone-dominated fovea, visible light OCT depicted two hyperreflective bands within band 4 (Fig. 1F). Here, aided by improved delineation of outer retinal layers in visible light OCT images (Figs. 1E, 1F and Supplement Video S1), we perform quantitative analysis of band 4 constituents in a cohort of normal eyes. Our detailed analysis forms the basis for a subsequent discussion of the reflectivity sources in band 4.

### System Description

A fiber-based, longitudinal chromatic aberration-corrected spectral/Fourier domain visible light OCT system, with rapid spectral shaping and axial motion tracking,^38^ was used in this study. A replaceable bite bar with three-dimensional translation was used to stabilize the subject during imaging. An external (fellow eye) fixation target consisting of an LED panel (2026, Adafruit Industries, New York, NY, USA) controlled by a Raspberry PI was used to adjust fixation over a 50° field-of-view. If needed, a prescribed corrective lens was provided for the fellow eye to enable the subject to focus on the LED panel.

### Imaging Protocol and Processing

**Figure 2.**
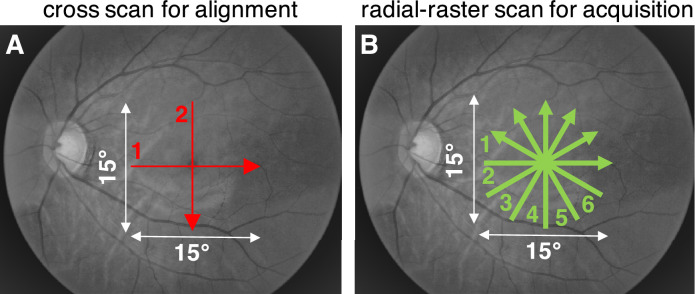
(**A**) First, a cross scan with visible red light and 0.01 to 0.02 mW incident power was used for alignment to the fovea centralis. (**B**) Then, a hybrid radial-raster scan with broadband visible light (shown as *green*) and 0.1 to 0.13 mW incident power was used for data acquisition. Approximate scan locations are shown on grayscale fundus photographs.

We first aligned with a cross scan with 0.01 to 0.02 mW incident power from 600 to 650 nm (Fig. 2A), attempting to locate the cross center on the foveola. Once centered, data were immediately acquired with 0.1 to 0.13 mW incident power^38^ from 500 to 650 nm (110 nm FWHM) at a 30 kHz scan rate. The incident power for image acquisition is two times lower than the maximum permissible exposures of the ANSI Z136.1–2014 and ANSI Z80.36-2016 American National Standard for Safe Use of Lasers published by Laser Institute of America.^41^^,^^44^^–^^46^ A hybrid radial-raster protocol with six angled raster scans, oriented every 30° in a spoke pattern, was used (Fig. 2B). The center of the hybrid radial-raster protocol coincided with that of the alignment cross scan. Each raster scan contained 840 fast axis axial scans across a 15° angular field of view, and 30 frames along the slow axis with a cumulative 0.15 mm offset for speckle reduction. Conventional OCT post-processing methods were applied,^47^ with water wavenumber calibration and transverse-dependent dispersion correction.^40^ Transverse (fast axis) and axial motion were corrected within each of the six raster scans before intensity averaging along the slow axis to yield six high-quality cross-sectional images that clearly depicted outer retinal bands (Fig. 1), with sub-degree precision.

The radial scan acquired topographic data across the macula in a reasonable time frame, avoided fixational drift observed with other protocols (such as a long and dense raster scan), and provided a clear foveal landmark in each scan to determine and correct for alignment error. However, a radial pattern has limitations, such as the lack of an en face image to assess centration or motion between radial scans. In light of these limitations, to thoroughly vet the results of this study, the data were analyzed in three different ways. First, each radial high-quality image was recentered so that the minimal inner limiting membrane (ILM) to band 1 thickness occurred at the image center (method 1). Second, the fovea was estimated as the location of minimal ILM to band 1 thickness across all six scans, and any relevant thickness data were interpolated in a new radial pattern centered at this location (method 2). Third, relevant thickness data were plotted against the inner band 2 to outer band 4 thickness (approximately, the outer segment, RPE, and BM combined), which normally decreases with increasing eccentricity and is therefore a suitable proxy for eccentricity (method 3). Because methods 2 and 3 helped to reduce centration errors inherent to the radial scan, results obtained via methods 2 and 3 are shown in the main study, unless otherwise noted. Reassuringly, all major results of the study were confirmed by all three methods. Comparisons between methods 1 and 2 are shown in Supplementary Figure S1.

### Human Subjects

Twenty-eight eyes of 19 human subjects without ocular pathology (21–57 years old) were imaged. The average age was 29.5 ± 7.4 years old with 10 males and nine females, and 12 whites and seven non-whites. Refractive errors ranged from −5D to 1.25D. The study was approved by the University of California, Davis Institutional Review Board and conducted in accordance with the tenets of the Declaration of Helsinki. Subjects were recruited for this research study at the University of California, Davis and informed consent was obtained before enrollment in the study. We elected to include only relatively young, normal eyes in this pilot study, thus excluding potential early age-related macular degeneration in the subject pool. Also, in younger eyes, melanin concentrates more apically and lipofuscin concentrates more centrally,^48^^,^^34^ whereas melanolipofuscin is relatively less common than in older eyes.^33^^,^^49^^,^^50^ The well-characterized, stereotyped laminar RPE organelle distribution in our cohort facilitates interpretation of the reflectivity profile of band 4, thus serving the overall goals of the study.

### Segmentation

**Figure 3.**
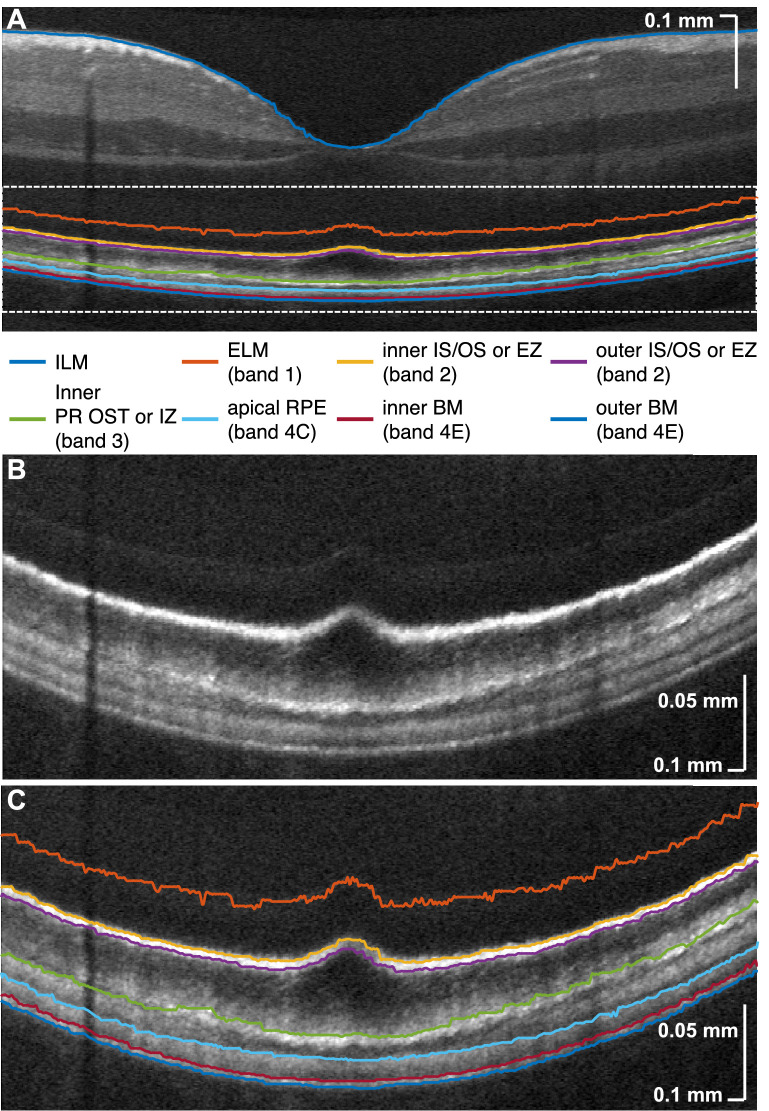
(**A**) Segmentation of outer retinal boundaries and ILM. Magnified view (**B**) shows that relevant outer retinal bands are well delimited by segmentation lines (**C**). ILM, inner limiting membrane; ELM, external limiting membrane; IS/OS, photoreceptor inner segment/outer segment junction; EZ, ellipsoid zone; PR OST, photoreceptor outer segment tips; RPE, retinal pigment epithelium; BM, Bruch's membrane. The IN•OCT numbering of the outer retinal bands^13^ is also given in the legend.

Eight boundaries were automatically segmented, using a variation of a prior approach,^9^ in each high-quality intensity image (Fig. 3A). Then, segmentation errors were manually corrected. Magnified views of the outer retina (Figs. 3B and 3C) illustrate that the segmentation lines delimit the major reflective bands, including the separation of BM and RPE, which has been challenging to visualize in eyes without pathology. Note that we opted to segment the inner boundary of the photoreceptor OST or IZ but did not attempt to distinguish between rod- and cone-associated OS layers, because their presence varies across the macula (Fig. 1F). In addition, the COST/CIZ band appeared to be more sensitive to pupil position than the ROST/RIZ band, as has been observed with NIR OCT.^51^ Thus, although the COST/CIZ inner boundary was reliably detected as the PR OST/IZ inner boundary in the fovea, occasionally the ROST/RIZ inner boundary was detected instead peripherally (see Fig. 3C and Supplementary Fig. S2).

### RPE Cell Body Thickness Measurement

In commercial NIR OCT (Fig. 1D), the normal human RPE presents as a part of a thick, hyperreflective band 4, where the cell body is indistinguishable from BM, the ROST, and the apical RPE processes. In visible light OCT,^38^^,^^52^ the RPE cell body is associated with a hyperreflective zone 4C and a hyporeflective zone 4D, which have not consistently been resolved by NIR OCT until recently.^19^ Previous work^25^ revealed that RPE multiple scattering from melanosomes and, potentially, melanolipofuscin is the main impediment to separate visualization of the RPE and BM bands and showed that shorter wavelengths improve the contrast between the two. Benefitting from this improved contrast, images acquired in this study readily distinguished these bands, enabling both morphometric analysis of the RPE cell body and investigation of its internal reflectivity for the first time.

### BM Thickness Measurement

BM is a pentalaminar structure consisting largely of collagen and elastin^53^ that mediates transport between the RPE and choriocapillaris. Although BM function is critical for retinal health, it is inconveniently situated beneath the melanosomes and melanolipofuscin of the RPE, which form a highly scattering wall that obscures OCT imaging of BM in pigmented subjects. Even at shorter wavelengths, RPE multiple scattering causes skewing and broadening of the BM intensity profile in OCT.^25^ Although these effects are insignificant in the context of quantifying the RPE thickness (see above), multiple scattering should be considered when quantifying the BM intensity profile.

**Figure 4.**
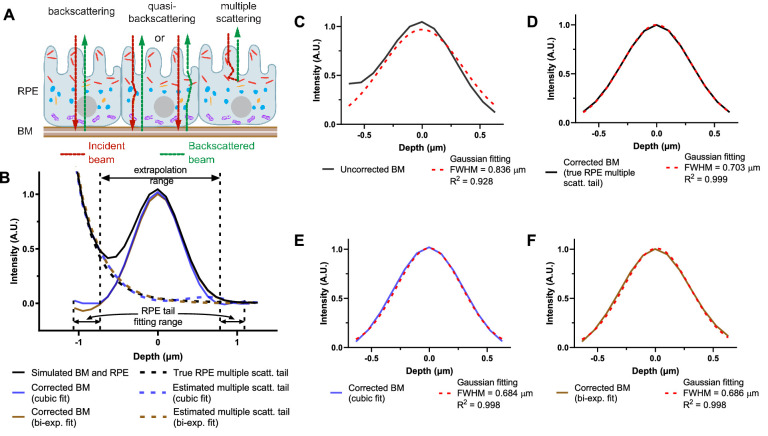
RPE multiple scattering affects BM thickness estimation. (**A**) Schematic diagram of granule distribution in RPE cells^8^^,^^34^ and the major light contributions of BM signal (backscattering from BM, quasi-backscattering from BM, and multiple RPE scattering) in OCT. (Melanosomes, *red*; melanolipofuscin, *orange*; lipofuscin, *blue*; mitochondria, *purple*; nucleus, *gray*.) Photoreceptor outer segments are not shown for simplicity. (**B**) Monte Carlo simulation^25^ (Supplementary Fig. S3) of visible light OCT including a BM reflection at 0 µm, RPE scattering, and the finite OCT full-width-at-half maximum (FWHM) axial resolution (1.0 µm in field or 0.707 µm in intensity). The RPE multiple scattering tail was extrapolated across the range shown by either cubic polynomial fitting (cubic fit) or biexponential fitting (bi-exp. fit). The corrected BM profile was then calculated by subtracting the extrapolated RPE multiple scattering tail from original simulated OCT signal. (**C**–**F**) Comparisons of BM FWHM thickness and R^2^ for standard Gaussian fitting with and without RPE multiple scattering correction. The FWHM of BM was overestimated without RPE multiple scattering correction (**C**) but was correctly estimated by subtracting the RPE multiple scattering tail (**D**), as determined from another simulation with a fully absorbing, nonreflective BM. Both cubic fitting (**E**) and biexponential fitting (**F**) performed well, with similar BM thickness estimates and R^2^ values. RPE, retinal pigment epithelium; BM, Bruch's membrane.

We can identify three major categories of detected light paths relevant to visualization of BM (Fig. 4A): (1) Light that travels to and from BM ballistically, backscattering/backreflecting off BM. (2) Light that forward scatters from the RPE before or after backscattering/backreflecting from BM. (3) Light that multiply scatters in the RPE. Because the OCT axial resolution cannot typically distinguish category 2 paths from category 1 paths,^25^ they are lumped together into “backscattered and quasi-backscattered light.” Light in category 3 is the most problematic for quantitative measurements of BM, because multiply scattering RPE tails skew and broaden the BM intensity profile (Fig. 4B), leading to an overestimation of BM thickness. Moreover, variability in RPE multiple scattering, possibly related to local variations in RPE granule content, could change the intensity profile, leading to spurious variations in BM thickness. Although improved at visible wavelengths,^25^ these potential problems are still present, necessitating a correction approach.

On the basis of a Monte Carlo simulation^25^ (Fig. 4B), which highlighted the limiting effects of RPE multiple scattering on BM quantification, we developed an approach to correct for RPE multiple scattering. The correction method was first validated in a Monte Carlo simulation (Supplementary Fig. S3), with a specular reflection representing BM (Figs. 4B–4F). Applying a simple Gaussian fitting approach, BM thickness was overestimated with a relatively poor goodness-of-fit (Fig. 4C). However, if the RPE multiple scattering tail was corrected by subtracting it from the intensity profile before Gaussian fitting, a BM FWHM thickness near the theoretical OCT intensity FWHM axial resolution (0.707 µm) is estimated, with an excellent R^2^ value. In silico, the RPE multiple scattering tail could easily be isolated by running another simulation with a fully absorbing, nonreflective BM (Fig. 4B, dotted line) and subtracted (Fig. 4D). Because it is not feasible to acquire an image without BM in vivo in the human eye, we instead devised an approach whereby the RPE multiple scattering tails were first estimated by fitting and then extrapolated across BM (see Fig. 4B for extrapolated region). In simulation, tail correction via both cubic polynomial and biexponential fitting for extrapolation improved the goodness-of-fit and yielded more accurate BM thickness estimates (Figs. 4E and 4F) than Gaussian fitting without tail correction (Fig. 4C).

To implement the RPE tail correction approach on in vivo OCT images, first the inner and outer boundary contours of BM were segmented, and axial scans were aligned according to the BM intensity peak between the inner and outer boundary contours. Next, transverse averaging and normalization were performed. The BM intensity profile was corrected by subtracting the RPE multiple scattering tail, which was estimated by extrapolating a fit across the BM region, as above. The FWHM thickness of BM was estimated by fitting a Gaussian function to the corrected BM intensity profile. Although cubic polynomial and biexponential fitting performed similarly for extrapolation of RPE tails across BM in silico, the relative performance of both methods was again assessed in vivo. The BM thickness data from the six radial angles were weighted by area to form thickness maps by sector and eccentricity.

## Results

### Topographical Analysis of BM Thickness

**Figure 5.**
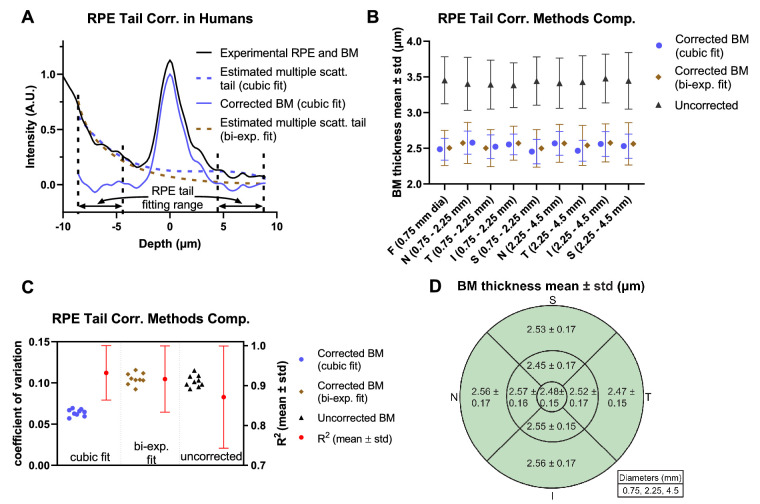
Topographical analysis of BM thickness. (**A**) Demonstration of RPE multiple scattering tail correction algorithm on visible light OCT data acquired in the human eye. Unlike simulations (Fig. 4) where the cubic fit and biexponential fit performed similarly, the cubic fit better represented in vivo intensity profiles from the human retina. (**B**) Average BM thicknesses with standard deviations across subjects in different macular areas, resulting from either no tail correction or correction via extrapolation of a cubic fit or biexponential fit. (**C**) Coefficient of variation (derived from B) and R^2^ values (means and standard deviations across subjects for the Gaussian fit to BM) for different correction methods. Extrapolating and correcting multiple scattering tails across the BM region with a cubic fit reduced both variability of estimated BM thickness (left axis) and error in the Gaussian fit of BM (right axis), from which BM thicknesses are derived (**C**). Therefore the tail extrapolation by a cubic fit was chosen as the preferred approach for subsequent quantification of BM. (**D**) Resulting BM thickness maps, summarizing data across all subjects. In the nine macular regions, average BM thickness ranged between 2.45 µm and 2.57 µm. RPE, retinal pigment epithelium; BM, Bruch's membrane; N, nasal; T, temporal; S, superior; I, inferior).

We first compared cubic polynomial and biexponential fitting for extrapolating RPE multiple scattering tails across BM. Note that both fits had four free parameters. Cubic polynomial fitting outperformed biexponential fitting in vivo, especially in regions with an elevated intensity outer to BM (Fig. 5A). This elevation could be caused by signal from choriocapillaris, which was not included in the Monte Carlo simulation. We quantitatively and topographically compared different tail correction methods against no tail correction in all subjects. Without tail correction, BM thickness was higher in all macular areas (3.38–3.48 µm) with higher standard deviations across subjects (Fig. 5B). Similar BM thickness estimates resulted from tail correction via a cubic polynomial fit (2.45–2.58 µm) and via a biexponential fit (2.50–2.58 µm). However, the biexponential fit resulted in higher standard deviations than the cubic polynomial fit in all macular areas (Fig. 5B). Accordingly, the BM thickness coefficient of variation resulting from the cubic polynomial fit (0.057–0.068) was markedly lower than the coefficient of variation resulting from the biexponential fit (0.092–0.12) and no correction (0.093–0.12) (Fig. 5C). Furthermore, the cubic polynomial fit for tail extrapolation and correction resulted in a higher Gaussian goodness-of-fit (R^2^ = 0.93 ± 0.07) than the biexponential fit (0.91 ± 0.08) and no correction (0.87 ± 0.13). Given the overall reduced variability and better description of the data observed in vivo, cubic polynomial fitting was chosen as the preferred method for tail extrapolation and correction. BM thicknesses estimated using this method are summarized in Figure 5D. No major topographical differences were noted across the macula (Supplementary Fig. S4). When averaged across the macula, BM thickness was found to be 2.50 ± 0.16 µm in our cohort, with individual subject measurements ranging from 2.09 to 3.05 µm. This range agrees with previous histological results in humans with a similar age distribution.^54^^,^^55^

### Topographical Analysis of RPE Thickness

**Figure 6.**
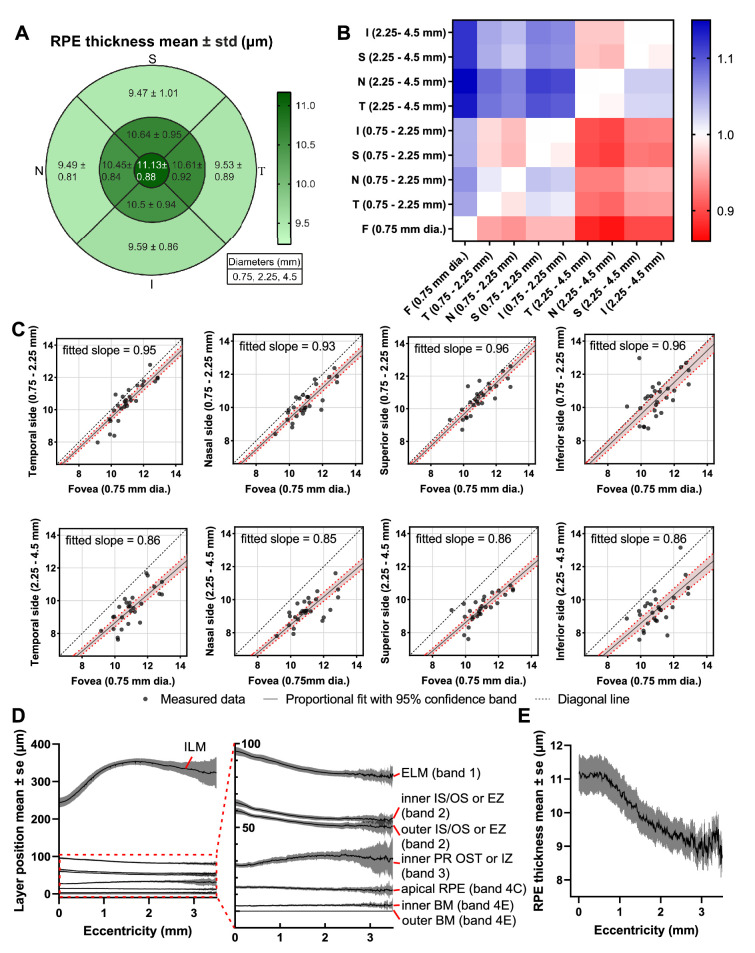
(**A**) Topographic mapping of macular RPE thickness. (**B**) Heatmap of RPE thickness comparisons between different macular areas. Constants of proportionality were determined via a proportional fit (i.e., a linear fit that passes through the origin) and are color-coded to indicate where the rows are smaller (*blue*) or larger (*red*) than the columns. (**C**) Detailed comparisons between fovea and other macular areas (first row labeled “F”) with 95% confidence band. (**D**) Retinal layer contours averaged across subjects and flattened to the outer BM depict topography relative to the foveal pit. (**E**) RPE thickness (mean with standard errors) shows a clear decreasing trend with eccentricity. RPE, retinal pigment epithelium.

The RPE thickness was taken as the distance between the apical RPE boundary and the inner BM boundary (Fig. 3). Topographical mapping of the RPE from all subjects with standard 0.75 mm, 2.25 mm, and 4.5mm circles showed that thickness was higher in the foveal 0.75 mm diameter disc (11.13 ± 0.88 µm) than in the 0.75 to 2.25 mm diameter annulus (10.55 ± 0.91 µm) and the 2.25 to 4.5 mm diameter annulus (9.56 ± 0.98 µm). We further compared RPE thicknesses between every pair of macular areas via a proportional fit (a linear regression through origin). The slopes of this fit, displayed as a heatmap (Fig. 6B), demonstrate a thicker RPE more centrally. In spite of the clear decrease with eccentricity, RPE thickness appeared to be circularly symmetric. A detailed expansion of the first row labeled “F” in Figure 6B (Fig. 6C) with proportional fits and 95% confidence bands when regressing against the foveal disc, confirms the decrease in RPE thickness between 0.75 to 2.25 mm (fitted slope from 0.93 to 0.96) and 2.25 to 4.5 mm (fitted slope from 0.85 to 0.86). The retinal contours, visualized by averaging across all subjects (Fig. 6D), summarize topographic relationships between outer retinal bands. RPE thickness (Fig. 6E) clearly exhibits a plateau in the fovea, accompanied by a decrease with eccentricity up to 3 mm.

**Figure 7.**
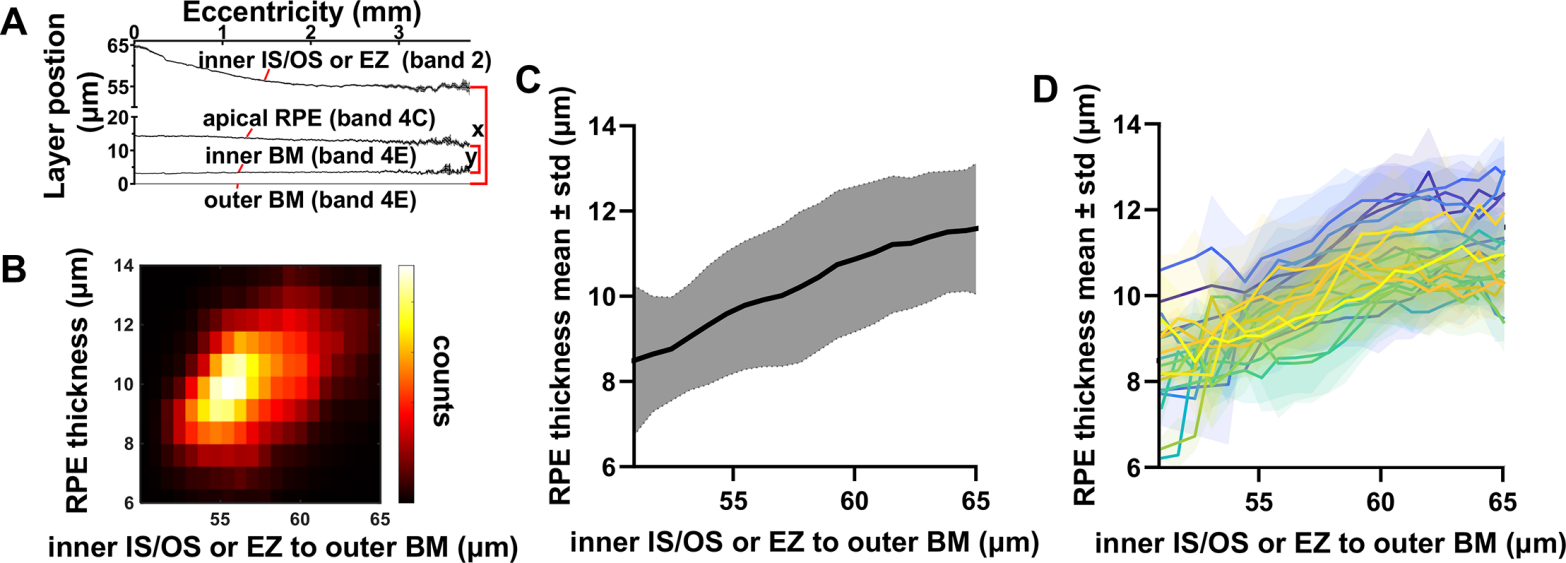
Centration-independent analysis of RPE thickness. (**A**) The distributions of the inner band 2 to outer band 4E (approximately, the combined outer segment, RPE, and BM thickness) and inner bands 4C to inner band 4E (RPE thickness) were summarized in a two-dimensional histogram (**B**). (**C**) Alternative representation of data in (**A**), showing mean RPE thickness and standard deviations across subjects. (**D**) Subject-by-subject RPE thicknesses with standard deviations show a similar trend. All results (**B**–**D**) support that RPE thickness increases with the distance from inner band 2 to band 4E. Because this distance is accepted to increase toward the fovea in normal eyes,^9^ this analysis indirectly confirms the decrease in RPE thickness with eccentricity in Figure 6. RPE, retinal pigment epithelium; BM, Bruch's membrane.

To circumvent the potential issue of radial scan centration errors, we performed an alternative analysis wherein we compared RPE thickness (inner band 4C to inner band 4E, y in Fig. 7A) to the combined outer segment, RPE, and BM thickness (inner band 2 to outer band 4E, x in Fig. 7A). The inner band 2 to band 4E thickness normally decreases from the fovea to the parafovea and perifovea^9^; therefore it serves as a suitable proxy for eccentricity in our subject cohort. The two-dimensional histogram (Fig. 7B), overall parametric plot (Fig. 7C), and the subject-by-subject parametric plots (Fig. 7D) all show that the two thicknesses increased together. This analysis, which does not require determination of eccentricity, confirms the result in Figure 6E that the RPE thickness decreases with eccentricity.

### RPE Internal Intensity Analysis

**Figure 8.**
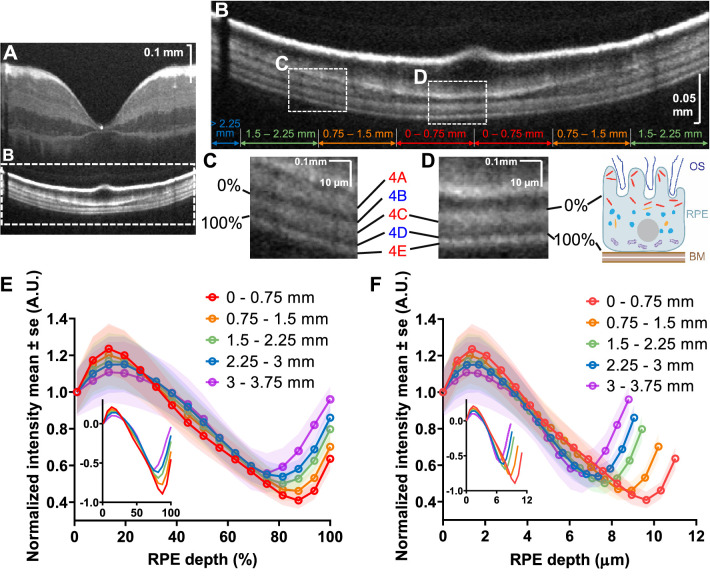
(**A**) RPE internal intensity was determined from the apical RPE boundary (0%) to the inner BM boundary (100%), and classified according to eccentricity (**B**), shown as radial distance from the fovea. Magnified views of foveal (**C**) and extrafoveal (**D**) regions, with the inner boundary of band 4C defining 0% of the RPE thickness, and inner band 4E defining 100% of the RPE thickness. Magnified views are shown alongside a schematic showing approximate granule distributions.^8^^,^^34^ (**E**) Normalized RPE interior intensity, averaged across subjects, shows a larger drop in intensity versus percentage RPE depth at smaller eccentricities. *Inset:* same data on a logarithmic scale. (**F**) However, when percentage RPE depth is converted to physical distance or absolute RPE depth (based on eccentricity-wise average RPE thickness across subjects), the higher foveal RPE thickness compensates the larger intensity drop, and logarithmic signal decay rates around 2 to 3 µm RPE depth are similar across eccentricities. *Inset*: same data on a logarithmic scale. RPE, retinal pigment epithelium; BM, Bruch's membrane

Clear delineation of the RPE borders by visible light OCT enabled a detailed analysis of the internal intensity of the RPE cell body (Fig. 8A). Inner band 4C was defined as 0% RPE depth (Fig. 8C), and inner band 4E was defined as 100% RPE depth (Fig. 8D), which is the entire RPE thickness. First, the image was classified into eccentricity ranges (Fig. 8A). The RPE internal intensity was calculated as a function of the percentage RPE depth. Intensities were averaged within each eccentricity range, normalized so the intensity at 0% RPE thickness was equal to 1, then averaged across subjects. RPE internal intensity was plotted versus percentage RPE depth (Fig. 8E) and absolute RPE depth (Fig. 8F), where each eccentricity range was assigned the subject-averaged thickness value. The higher attenuation across the RPE in the fovea (Fig. 8E) may imply that the en face areal density of scattering and absorbing granules is higher. However, similar attenuation rates versus depth within the apical RPE across eccentricities (appreciated on the log scale inset in Fig. 8F) suggest that the volumetric density of attenuating granules may be more similar across eccentricities. In other words, the larger drop in the OCT intensity across the RPE in the fovea could be explained, at least partially, by greater foveal RPE thickness (Fig. 6E) and the resulting higher attenuation.

## Discussion

In the ensuing discussion, we adopt the IN•OCT terminology “zone” for OCT features that seem to localize to a particular anatomic region.^13^ As described above, we consistently find that visible light OCT delineates five zones within band 4, which we enumerate as 4A to 4E (Fig. 1). Below, we discuss each zone, highlighting how our data aid the interpretation of each. We also use the terms hyporeflective and hyperreflective to describe OCT intensity image appearance, using more physically precise terminology where appropriate.

### Zone 4A

Visible light OCT depicted a hyperreflective band just outer to the CIZ/COST. This band is often known alternatively as the ROST or RIZ. Candidate reflectivity sources include the refractive index discontinuity between the rod tips and extracellular space consisting of the interphotoreceptor matrix, and the melanosomes in the apical RPE processes. The observation that the ROST hyperreflective band appears in mice,^56^ rats,^57^ and humans^58^ with albinism shows that melanin is not required for reflectivity of this band, but this observation does not exclude possible contributions from melanosomes in the apical processes. In our cohort, the hyporeflective gap between the ROST/RIZ band and COST/CIZ bands was not uniformly visible, and the ROST band unequivocally disappeared in the fovea. Thus no attempt was made to segment the inner boundary of this band, although it was occasionally visualized with stark relief (e.g., Fig. 8C).

### Zone 4B

Visible light OCT consistently revealed a hyporeflective region outer to the COST/CIZ in the fovea and band 4A at larger eccentricities, designated as zone 4B. It has been suggested^8^ that this hyporeflective zone could correspond to a thin region, observed histologically in primates,^59^^,^^60^ that is devoid of melanosomes, between the apical processes and the melanosomes in the RPE cell body. This suggestion seems plausible, regardless of whether the reflectivity sources in band 4A are the rod outer segment tips, melanosomes, or both. Importantly, segmentation of this zone enabled morphometry of RPE by providing a reasonable reflectivity-based marker of the RPE soma inner aspect.

### Zone 4C

Visible light OCT depicted a thick hyperreflective band outer to zone 4B. Although this band is typically assumed to represent the RPE, the reflectivity sources remain a matter of debate.^61^ With the 1.0 µm resolution of visible light OCT, we provide a detailed quantitative description of this purported RPE band, which is assumed to encompass zones 4C and 4D. These measurements show a thickening towards the fovea, in agreement with the histologically observed lengthening of the RPE cells in the fovea.^48^ Second, the reflectivity of band 4C decreased from the inner aspect to the outer aspect (near the hyporeflective zone 4D).

### Zone 4D

Visible light OCT revealed a hyporeflective zone of variable thickness outer to zone 4C. Because our measurements of RPE thickness that included this zone were found to be consistent with prior histological accounts,^48^ we are compelled to hypothesize that this hyporeflective zone belongs to the RPE. Because the reflectivity is graded and hyporeflective zone 4D is very thick compared to the basal lamina and RPE plasma membrane, it is reasonable to assume that this hyporeflective zone coincides with the basal RPE soma.

Because organelles are responsible for large variations in intracellular refractive index^26^^,^^62^^–^^64^ on a spatial scale that can generate backscattering, we look to organelle distributions to explain OCT signal. Among organelles that might be relevant for OCT,^8^ the graded apical-to-basal RPE signal intensity profile reported here most resembles the distribution of melanosomes, followed by melanolipofuscin, lipofuscin, and mitochondria (in order of most to least similar).^34^ In particular, it has been shown that mitochondria localize more basally near the cell nucleus,^8^ whereas melanosomes are less numerous and melanin content is lower.^48^ The low reflectivity of the basal RPE, where mitochondria congregate, seems to argue against the hypothesis that mitochondria are a dominant reflectivity source in the RPE, at least in visible light OCT.

Although it is tempting to equate OCT intensity (reflectivity) and backscattering, both attenuation and multiple scattering effects should be considered when interpreting OCT intensity profiles. First, we expect that apical melanosomes attenuate light transmitted to and backscattered from the basal RPE, thus making the basal RPE appear more hyporeflective. Even so, the basal RPE is still less reflective than BM, which is similarly subject to melanosome attenuation. Second, the RPE internal intensity is concave downward on a log scale near the apical-basal transition (insets in Figs. 8E and 8F), before reaching a nadir in the basal RPE and increasing again near BM. Because OCT intensity in a uniform medium decreases linearly on a log scale, such an intensity profile supports lower intrinsic backscattering in the basal RPE than the apical RPE. Third, our previous work suggested that multiple light scattering by melanosomes creates a spurious signal that mimics backscattering from basal RPE.^25^ Thus in pigmented subjects, the true, intrinsic backscattering of the basal RPE is likely even lower than depicted on OCT images. This assertion is further supported by comparisons of pigmented and albino mice in other studies.^25^ Thus, even after considering attenuation and multiple scattering, our results still suggest that intrinsic backscattering from organelles in the basal RPE is low.

In summary, we conclude that the contribution of the basal RPE to band 4 intensity is less than that of BM and the apical RPE. We propose that mitochondria should be ranked, at highest, third, behind melanosomes and BM, as a reflectivity source in band 4. This empirical statement about the reflectivity of mitochondria relative to other band 4 structures, is not necessarily in conflict with the well-established fact that mitochondria can and do scatter light.^65^^,^^66^ The refractive index of the surrounding medium and nearby organelles (i.e., packing effects) can also play a role in light scattering.

### Zone 4E

Visible light OCT consistently revealed a distinct hyperreflective band outer to the RPE. This band has uniformly been attributed to Bruch's membrane (BM) in the literature.^9^^,^^31^ Although Bruch's membrane is a five-layered structure,^53^ the majority of its thickness is accounted for by the inner collagenous, elastic, and outer collagenous layers (layers 2–4).^6^ Because the RPE basal lamina (layer 1 of BM) is only 0.14 to 0.15 µm thick,^67^ the inner boundary of the BM band on OCT, representing hyperreflective connective tissue, is a reasonable morphometric marker for the outer RPE aspect.

The present work highlights that the OCT intensity profile near BM depends critically on multiple scattering from the melanosomes inner to BM. Our results are fully consistent with those of Wilk et al.,^68^ who noted that the absence of RPE melanin “unmasks” the BM band in subjects with albinism. The present work extends the observations of Wilk et al.^68^ by clarifying that multiple scattering in melanin-containing melanosomes is responsible for obscuring BM. We further suggest that visible light OCT may help to unmask BM, even in subjects without albinism, by improving the contrast of BM relative to the hyporeflective basal RPE.^25^ By explicitly accounting for the effects of multiple scattering by fitting, our biophysically-validated method of quantifying BM width reduces variability of BM measurements.

One limitation of this study is the relatively small subject cohort. As mentioned above, although the young age of the cohort greatly facilitated interpretation of the data, future studies will need to examine RPE reflectivity distributions in the eyes of older subjects and in diseases such as age-related macular degeneration, where organelle distributions change markedly.^69^^,^^70^ We found no statistically significant correlation between either age or prescription and either BM or RPE thickness. We also did not detect statistically significant differences between BM and RPE thickness in males versus females. In addition, although the major retinal reflectivity sources are not expected to differ for visible light OCT and NIR OCT, relative backscattering of structures with different sizes may change at shorter visible wavelengths. Errors in thickness measurements on the order of a few percent are expected because of differences between the water refractive index assumed in reconstruction and the true tissue refractive index. Errors may be large in the RPE, which has a high lipid and melanin content; however, topographic trends in thickness can still be assessed with more confidence, under the assumptions that refractive indices do not change with eccentricity. Finally, our measurements of BM might be overestimated because of the convolution of a Gaussian intensity PSF with the true axial intensity profile. Assuming that the true intensity profile is Gaussian and the convolution occurs in intensity, we find that a measured BM thickness of 2.50 µm must be corrected to $\sqrt{2.50^{2}-0.71^{2}}=2.40$ µm. Assuming that the true intensity profile is rectangular, no correction is required. Additionally, when considering the possible effects of melanosome translocation,^71^ if any, it is important to stress that the retina being imaged by visible light OCT is light adapted in this study. Finally, for validation, this study compared subject-averaged in vivo results to trends in separate cohorts assessed ex vivo by electron^8^^,^^33^ and autofluorescence^34^ microscopy. Validation of our findings on a subject-by-subject basis with corresponding histology remains a topic for future work.

## Conclusions

This study provides a detailed subcellular account of outer retinal reflectivity in visible light OCT. With our proposed assignment of outer retinal bands, measurements of the RPE and BM are consistent with histology. RPE internal reflectivity suggests that signal originates from apical organelles. Our study can further serve as a quantitative benchmark to assess alternative attributions of outer retinal bands.

## Supplementary Material

Supplement 1

Supplement 2
