# Supplementary material for: Visible Light Optical Coherence Tomography (OCT) Quantifies Subcellular Contributions to Outer Retinal Band 4
Source: Transl Vis Sci Technol. 2021 Mar 26;10(3):30. doi: 10.1167/tvst.10.3.30 (PMC7998011; doi:10.1167/tvst.10.3.30)
Supplement: Supplement 2 [file tvst-10-3-30_s002.pdf]

Supplemental Materials

1. Comparison of methods for correcting radial scans.

For our hybrid radial-raster scan protocol (Figure 2B), an important consideration is foveolar centration. After data acquisition, centration errors can be corrected in slightly different ways by methods 1 (angle-by-angle correction) and 2 (global scan protocol correction), as described in the manuscript. The outer retinal layer contours recovered by method 1 (Figure S1A) show similar trends as the layer contours recovered by method 2 in the main manuscript (Figure 6D), albeit with less sharp foveal features. For instance, the ILM layer contour recovered by method 2 shows a more prominent foveal pit (Figure S1B), and agrees more with normative values[1] of retinal thickness from other studies. Hence method 2 was chosen for the majority of the results in the main manuscript.

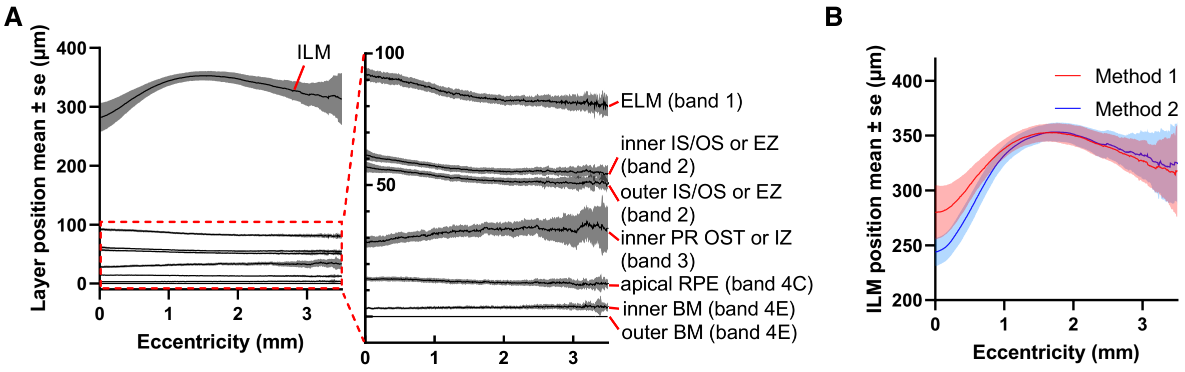

Figure S1. (A) Retinal layer contours recovered by method 1, averaged across subjects and referenced to BM at 0 microns. (B). Comparison of ILM contours shows that the foveal pit recovered by method 2 is deeper than that recovered by method 1.

2. Rod and Cone PR OST or IZ bands vary with eccentricity.

The appearance of COST/CIZ and ROST/RIZ varied with eccentricity (Figure S2). COST/CIZ was always well-visualized in the fovea. However, occasionally, COST visualization was diminished

in the perifovea (white arrow in zoom). On the other hand, ROST/RIZ was never seen in the foveola, with visualization improving from the parafovea to the perifovea. These observations can be explained by the photoreceptor distribution, which transitions from a cone-dominated fovea to a rod-dominated periphery[2], and the greater sensitivity of waveguiding in peripheral cones, relative to other photoreceptors, to incident light angle. Because of these issues, separation of rod from cone photoreceptor outer segment tips was challenging in some data sets, and was not pursued further in this study. Variable peripheral COST visibility was reflected in the high variability of the inner band 3 contour (Figure 6D and Figure S1A).

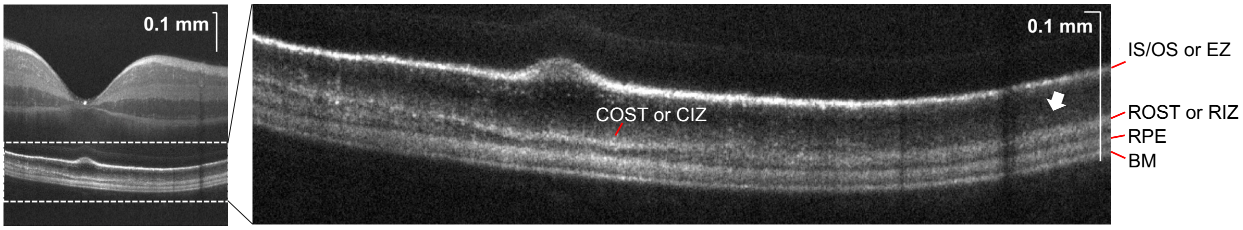

Figure S2. Human retinal image with zoom of the outer retina. As shown by the white arrow, the COST or CIZ band disappears on the right hand side of the image, whereas the ROST or RIZ band remains visible. This may result from heightened sensitivity of the COST band to incident light angle[3].

3. Monte Carlo simulation parameter table for assessing RPE multiple scattering effects.

A Monte Carlo simulation model (Figure S3A) with realistic RPE parameters[4] (Figure S3B) was employed in this study to investigate the effects of RPE multiple scattering on BM visualization[4]. Scattering was confined to a melanosome band (4C) in the apical RPE, while BM (band 4E) was generated by a Fresnel reflection at the interface between the basal RPE and a medium below with a slightly different refractive index and a medium with an absorbing lower boundary. A 6 μm diameter, collimated incident beam and an identically sized detector with a 2.5 degree polar acceptance angle were used in simulation. OCT intensity profiles versus depth were created from the weighted photons and their times-of-flight, assuming a refractive index of 1.47 and performing

Gaussian binning of photons to achieve an OCT axial resolution of 1.0 microns (0.71 microns in intensity). This simulation incorporated several simplifying assumptions, including a specular reflection for BM, an infinitesimally small true BM thickness, and no scattering from the basal RPE or the choriocapillaris. Also, rather than assume the RPE refractive index as in this simulation, *in vivo* OCT images in the main manuscript were reconstructed assuming a water medium. In spite of these differences, the simulation provided a useful preliminary tool to assess methods for estimating BM thickness.

A

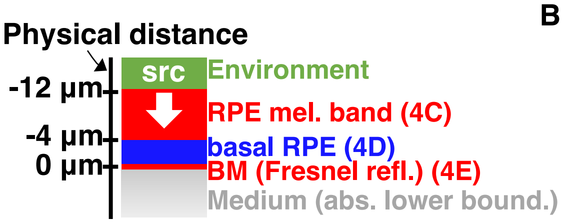

B

n	μ_a (mm^{-1})	μ_s (mm^{-1})	g
1.43	-	-	-
1.47	139.4	112.5	0.84
1.47	0	0	-
1.467	0	0	-

Figure S3. (A) Monte Carlo simulation model (src.: source beam; mel.: melanosome; refl.: reflection; abs.: absorbing). (B) Monte Carlo simulation parameters used in Figure 4 to assess RPE multiple scattering. Hyper-reflective bands (zones 4C and 4E) are shown in red, and the hypo-reflective basal RPE zone (4D) is shown in blue.

4. Pair-wise BM thickness comparison

Similar to our analysis of RPE thickness in the main manuscript (Figure 6), we performed pair-wise BM thickness comparisons of different macular regions (Figure S4). Relative to the foveal region, other macular regions showed no major differences in BM thickness (diagonal line was within 95% confidence band for the proportional fit) (Figure S4A). The heatmap of BM thickness comparisons between macular areas further supported this conclusion (Figure S4B). While the nasal 2.25-4.5mm and foveal regions tended to be slightly thinner and the nasal 0.75-2.25 mm region tended to be thicker, further investigation with more subjects is required to confirm these observations.

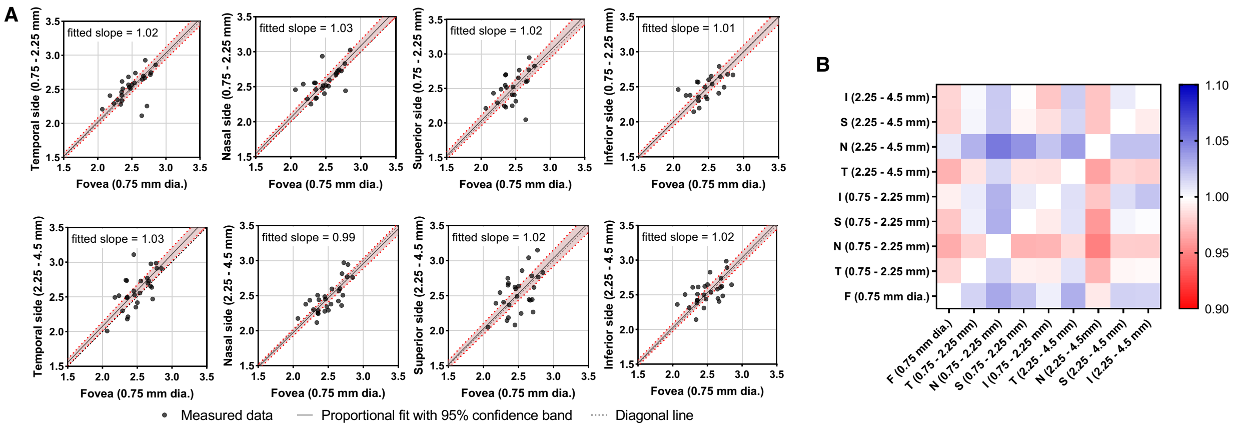

Figure S4. (A) Detailed comparisons between BM thickness in the fovea and other macular areas (proportional fit with 95% confidence band). (B) Heatmap of BM thickness comparisons between different macular areas shows that average topographic variations are typically on the order of a few percent, significantly less than RPE variations (Figure 6B).

1 Sull, A. C. *et al.* Comparison of spectral/Fourier domain optical coherence tomography instruments for assessment of normal macular thickness. *Retina* **30**, 235-245, doi:10.1097/IAE.0b013e3181bd2c3b (2010).

2 Curcio, C. A., Sloan, K. R., Kalina, R. E. & Hendrickson, A. E. Human photoreceptor topography. *J Comp Neurol* **292**, 497-523, doi:10.1002/cne.902920402 (1990).

3 Gao, W., Cense, B., Zhang, Y., Jonnal, R. S. & Miller, D. T. Measuring retinal contributions to the optical Stiles-Crawford effect with optical coherence tomography. *Opt Express* **16**, 6486-6501, doi:10.1364/OE.16.006486 (2008).

4 Zhang, T. *et al.* Visible light OCT improves imaging through a highly scattering retinal pigment epithelial wall. *Optics Letters* **45**, 5945-5948, doi:10.1364/OL.405398 (2020).
